# Characterization of the genes responsible for rubber degradation in *Actinoplanes* sp. strain OR16

**DOI:** 10.1007/s00253-020-10700-1

**Published:** 2020-07-18

**Authors:** Namiko Gibu, Tomoka Arata, Saya Kuboki, Dao Viet Linh, Masao Fukuda, Alexander Steinbüchel, Daisuke Kasai

**Affiliations:** 1 Department of Bioengineering, Nagaoka University of Technology, Nagaoka, Niigata 940-2188 Japan; 2 Department of Biomedical Engineering, National University of Singapore, Singapore, Singapore; 3 Department of Biological Chemistry, Chubu University, Kasugai, Aichi 487-8501 Japan; 4 Institut für Molekulare Mikrobiologie und Biotechnologie, Westfälische Wilhelms-Universität Münster, Münster, Germany; 5 Environmental Science Department, King Abdulaziz University, Jeddah, Saudi Arabia

**Keywords:** *Actinoplanes*, Complete genome sequence, Natural rubber degradation, TetR/AcrR-type transcriptional regulator

## Abstract

A Gram-positive rubber-degrading bacterium, *Actinoplanes* sp. strain OR16 (strain NBRC 114529), is able to grow on agar plates containing natural and synthetic rubber as the sole sources of carbon and energy. When this strain was grown on natural rubber latex overlay agar plates, translucent halos around the cells were observed. To identify the natural rubber degradation genes and other features of its metabolism, its complete genome sequence was determined. The genome of OR16 consists of 9,293,892 bp and comprises one circular chromosome (GenBank accession number AP019371.1) with a G + C content of 70.3%. The genome contains 8238 protein-coding and 18 rRNA genes. A homology search of the genome sequence revealed that three genes (*lcp1*, *lcp2*, and *lcp3*) are homologous to an extracellular latex-clearing protein (Lcp) of *Streptomyces* sp. K30. RT-PCR analysis revealed that *lcp1* and *lcp2* seem to constitute an operon. Purified *lcp* gene products have oxygen consumption activity toward natural rubber latex, suggesting that all these genes encode rubber-degrading enzymes in OR16. Quantitative reverse transcription-PCR analysis indicated that the transcription of these genes is induced during the growth of OR16 on natural rubber. The genes located adjacent to *lcp1* and *lcp3*, which code for a TetR/AcrR-type transcriptional regulator, can bind to the promoter regions of these *lcp* genes. It is suggested that the putative regulators play a role in regulating the transcription of the *lcp* genes. These results strongly suggested that three *lcp* genes are required for the utilization of natural rubber in strain OR16.

**Table:** 

**Key Points** *• The complete genome sequence of Actinoplanes sp. strain OR16 was determined.* *• Three lcp genes which are involved in the natural rubber degradation in OR16 were identified.* *• Transcription of these lcp genes is induced during utilization of rubber in OR16.* *• Two regulators, which bind to the promoter regions of lcp, were determined.*

**Electronic supplementary material:**

The online version of this article (10.1007/s00253-020-10700-1) contains supplementary material, which is available to authorized users.

## Introduction

Natural rubber (NR) is a biopolymer containing poly(*cis*-1,4-isoprene) as a main component and is produced by over 2000 plants including *Hevea brasiliensis* (Backhaus 1985). NR is used globally in industry for tires, seismic isolation rubbers, medical gloves, and many other products. The waste of these products is permanently disposed and treated by combustion or landfill processes, which are hazardous to the environment. It is necessary to find an alternative treatment process for rubber waste. NR-degrading microorganisms, including Gram-positive and Gram-negative bacteria, have been identified and characterized in the past 20 years (Linos et al. 2000a; Linos et al. 2000b). Because of their rubber degradation ability, these bacteria are expected to be used as practical tools for the treatment process of rubber waste.

Microbial degradation of NR has been widely reported in Gram-positive bacteria such as *Streptomyces*, *Gordonia*, *Rhodococcus*, and *Nocardia* species and in only a few of Gram-negative bacteria including *Steroidobacter cummioxidans* 35Y and *Rhizobacter gummiphilus* NS21^T^ (Bröker et al. 2008; Imai et al. 2011; Jendrossek and Reinhardt 2003; Sharma et al. 2018). Two major types of NR-degrading enzymes, rubber oxygenase (RoxA/RoxB) and latex-clearing protein (Lcp), have been characterized in Gram-negative and Gram-positive bacteria, respectively. Exception includes a Gram-negative bacterium, *Solimonas fluminis*, which has an *lcp* homologous gene (Birke and Jendrossek 2019). RoxA from strain 35Y is an *exo*-type diheme oxygenase that oxidizes poly(*cis*-1,4-isoprene) into the C_15_ tri-isoprenoid product 12-oxo-4,8-dimethyltrideca-4,8-diene-1-al (Braaz et al. 2004). On the other hand, RoxB degrades NR via *endo* cleavage of poly(*cis*-1,4-isoprene) (Birke et al. 2017). It has been reported that the two rubber oxygenases are involved in the NR degradation in Gram-negative bacteria including several myxobacteria (Birke et al. 2017). Recently, it has been reported that the functional RoxB and RoxA orthologs, LatA1 and LatA2, respectively, are essential for NR degradation by *R*. *gummiphilus* NS21^T^ (Birke et al. 2018; Kasai et al. 2017).

**Fig. 1:**
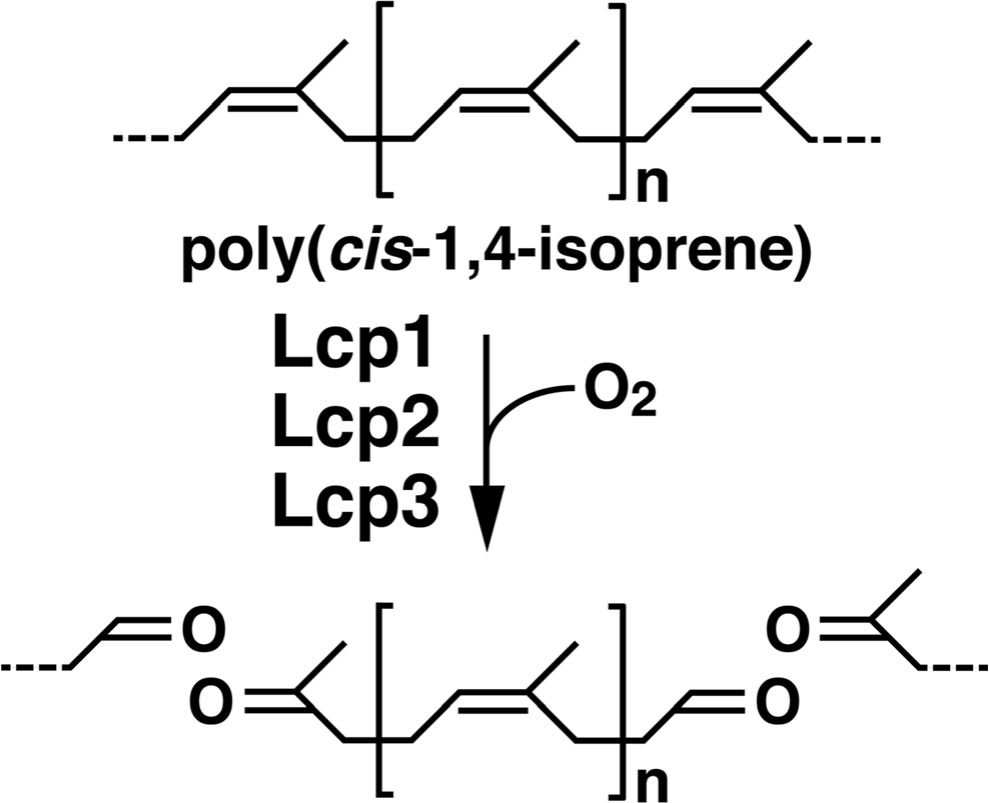
Proposed cleavage reaction for poly(*cis*-1,4-isoprene) by Lcps. Poly(*cis*-1,4-isoprene) is cleaved to form the products with aldehyde and keto end groups

The NR-degrading actinomycete, *Streptomyces* sp. strain K30 has an Lcp, which is a cofactor-independent oxygenase that catalyzes the oxidative *endo* cleavage of poly(*cis*-1,4-isoprene) into low-molecular-weight products containing aldehyde and keto end groups (Fig. 1) (Birke and Jendrossek 2014; Bröker et al. 2008). In contrast to Gram-negative rubber degraders, almost all rubber-degrading actinomycetes, including *Streptomyces*, *Nocardia*, and *Rhodococcus* species, except for *Gordonia polyisoprenivorans* VH2, have a single *lcp* homolog (Hiessl et al. 2012; Luo et al. 2014; Oetermann et al. 2018; Watcharakul et al. 2016).

Recently, a rubber-utilizing bacterium, strain OR16 (strain NBRC 114529), was isolated from a botanical garden in Japan (Imai et al. 2011). This strain is capable of growing on solid media containing NR or synthetic isoprene rubber (IR) and produces translucent halos on NR latex overlay agar plates. Taxonomic analysis revealed that strain OR16 belongs to the genus *Actinoplanes*. The genus *Actinoplanes* contains the rubber-degrading strains *A. missouriensis* NBRC 102363, *A. italicus* DSM 43146, and *A. utahensis* NBRC 13244 (Jendrossek et al. 1997); however, all the genes and their gene products responsible for rubber degradation have not yet been characterized genetically and biochemically. Here, the genome sequence analysis of strain OR16 identified three *lcp* homologous genes which are responsible for the rubber utilization of this strain. Multiple *lcp* genes were characterized in *G. polyisoprenivorans* VH2, but the two *lcp* genes are located in chromosome and plasmid. On the other hand, three *lcp* genes of strain OR16 were located on the chromosome. Recently, three *lcp* homologous genes have been identified in *Streptomyces* sp. strain CFMR7 by the genome sequence analysis (Nanthini et al. 2017). However, the functions of the genes have not been characterized at the molecular level, and the roles of their gene products are not clear. To gain insight into the rubber degradation system including three *lcp* homologous genes and the characterization of the functions of multiple *lcp* genes of *Actinoplanes* sp. strain OR16 is important. This is the first report of the functional characterization of rubber-utilizing bacterium which has three *lcp* genes.

## Materials and methods

### Bacterial strains, plasmids, and culture conditions

*Actinoplanes* sp. strain OR16 and its mutant derivatives were routinely grown at 30 °C in PYM medium (0.5% Bactopeptone, 0.3% yeast extract, and 0.1% MgSO_4_•7H_2_O; pH 7.0) or W minimal salt medium (Araki et al. 2011) containing 10 mM sodium succinate. Growth on NR or IR was examined using rubber overlay agar plates. To prepare the rubber overlay agar plates, W agar medium containing deproteinized NR (Chaikumpollert et al. 2012) or synthetic isoprene rubber at a final concentration of 0.4% (*v*/v) was overlaid to form a thin layer on a solid medium. *Escherichia coli* strains were cultivated aerobically at 30 °C or 37 °C in LB medium. If necessary, ampicillin (100 mg/l), kanamycin (25 mg/l), chloramphenicol (25 mg/l), tetracycline (10 mg/l), and apramycin (100 mg/l) were added to the medium.

### DNA manipulations, nucleotide sequencing, and sequence analysis

DNA manipulations including total DNA isolation and nucleotide sequencing were performed as previously described (Masai et al. 1995). Analysis of nucleotide sequences was carried out as previously described (Kasai et al. 2009). The genome sequence of OR16 was determined by the combination of MiSeq, PacBio RS II system, and Sanger sequencing. These sequencing data were assembled by CLC genomics workbench (Qiagen). Annotation was performed using NCBI Prokaryotic Genome Annotation Pipeline ver.3.1 (Tatusova et al. 2016) and RAST server (Aziz et al. 2008). Genome sequences of strain OR16 and of related *Actinoplanes* spp. were compared by calculating average nucleotide identity (ANI) values using the JSpecies Web Service (Richter et al. 2016). Tat signal sequences were predicted using the TatP 1.0 software (http://www.cbs.dtu.dk/services/TatP/) (Bendtsen et al. 2005).

### Expression of *lcp* genes in *E. coli*

The coding regions of *lcp1*, *lcp2*, and *lcp3* were independently amplified by PCR using lcp1_F and R, lcp3_F and R, and lcp2_F and R primer pairs (Table 1). Each forward primer contained an *Nde*I site at the start codon of the corresponding target gene. The amplified fragments were separately cloned in pJET1.2 to generate the *Nde*I fragment containing the entire target gene. The *Nde*I fragments containing either of *lcp* genes were individually cloned in the expression vector, pET23b. The resultant plasmids were independently introduced into *E. coli* Rosetta-gami B(DE3)pLysS, and the transformants were grown at 30 °C in LB medium containing kanamycin, chloramphenicol, and tetracycline. When the absorbance at 600 nm (*A*_600_) of the culture reached 0.5, 1 mM isopropyl-β-D-thiogalactopyranoside was added, and the cultures were further incubated at 20 °C for 16 h. The resulting cells were harvested and resuspended in 50 mM Tris-HCl (pH 7.4). The cell extract was prepared by using French pressure cell press. After centrifugation, the resulting supernatant was applied to a Ni Sepharose 6 Fast Flow column (GE Healthcare, Buckinghamshire, UK) previously equilibrated with buffer A consisting of 50 mM Tris-HCl (pH 7.5), 500 mM NaCl, and 100 mM imidazole. Proteins were allowed to bind for 1 min at 4 °C while rotating, followed by washing five times in 5 ml of buffer A. His-tagged proteins were eluted with 5 ml of buffer B consisting of 50 mM Tris-HCl (pH 7.5), 500 mM NaCl, and 500 mM imidazole, and the fractions were pooled and concentrated.

**Table 1:** Primer sequences used in this study

Oligo nucleotide	Sequence (5′ to 3′)
lcp1_F	CATATGGAACCAATGAGCAGGCG
lcp1_R	CTCGAGGGTCGGGCGGTTGGTG
lcp2_F	CATATGAAACGCAGAGTTCTGCTGTCC
lcp2_R	AAGCTTTTCGGGCCTGTTGATGG
lcp3_F	CATATGCAAAATCTCAGCAGACG
lcp3_R	GAGCGCTGACAAGCTTGGCG
lcp1_lcp2_F2	CGACATCATGGTCACCTGGCACAG
lcp1_lcp2_R2	CCCTTGGAGTAGTAGACCGACCAG
lcp2_oxiB_F2	GATCATCAGCCAGGAGGACATTCTC
lcp2_oxiB_R2	GTCGAGGTCCATCGCGAAGGTCTTG
oxiB_oxiA_F2	CAGTTCAGCCATACATCGCCGTACC
oxiB_oxiA_R2	TTGAGATGACTGGTGCACGCCC
QRTlcp1_F	GCGGACCTGGAAGAAGAAC
QRTlcp1_R	TAGAGGACGCCGAGGTAGAG
QRTlcp1_Probe	CCGTCGACTTCAACGAGAAG (FAM-TAMRA)
QRTlcp2_F	GAGGCCTGGTCGGTCTACTA
QRTlcp2_R	CCAGGTGACGAGAATGTCCT
QRTlcp2_Probe	GTGACTGCGTCGTCAACG (FAM-TAMRA)
QRTlcp3_F	CGTACGGCTACGACCTCAGT
QRTlcp3_R	GTTGCTGATCGGGATGAAGT
QRTlcp3_Probe	CGTCACCTCGAACAAGACG (FAM-TAMRA)
QRT16sOR16_F	AGCGTTGTCCGGATTTATTG
QRT16sOR16_R	CCTCCTGATATCTGCGCATT
QRT16sOR16_Probe	AGGCTAGAGTTCGGTAGGGG (FAM-TAMRA)
tetR_lcp1_F	GGCGCCGAGTGAATCCATCG
tetR_lcp1_R	CGATGTAAGGAGACCGGCAT
lcp3_UP_F	CAGCGGAGCGAGCTGCATGC
lcp3_UP_R	CATTCGCTTACCTACTTACG

### Enzyme assays

The activities of Lcp1, Lcp2, and Lcp3 were assayed by measuring the substrate-dependent oxygen consumption rate. Each 2-ml assay mixture contained 50 mM Tris-HCl buffer, NR (final concentration was 0.2%), and purified enzyme (25 μg of protein). The reaction mixture was incubated at 30 °C, and the oxygen consumption rate was determined with an oxygen electrode (Dual Digital Model 20; Rank Brothers Ltd., Cambridge, England). One unit of enzyme activity was defined as the amount of activity that resulted in consumption of 1 μmol of O_2_ per 1 min. Specific activity was expressed in units per milligram of protein. The optimal pH and optimal temperature for Lcp1, Lcp2, and Lcp3 were determined at pH and temperature ranges of 6.0 to 8.5 and 25 to 40 °C, respectively, by using 50 mM Tris-HCl buffer.

### Quantitative reverse transcription-PCR (qRT-PCR) analysis

The cells of OR16 were grown on a NR-overlay agar medium or W agar medium containing 10 mM sodium succinate at 30 °C for 3 days. They were collected using centrifugation and washed with 0.9% NaCl. The total RNA was extracted from the cells with ISOGEN II (Nippon Gene Co., Ltd., Tokyo, Japan) according to the manufacturer’s instructions and was treated with RNase-free DNase I (Roche). Single-stranded cDNA was synthesized from 1 μg of total RNA with 100 U of PrimeScript II reverse transcriptase (Takara Bio Inc., Otsu, Japan) and random hexamer primers in a 30 μl reaction mixture. The cDNA was subjected to RT-PCR and qRT-PCR analyses. These PCR analyses were carried out using the specific primers (Table 1) according to the previous reports (Kasai et al. 2017).

### Electrophoretic mobility shift assays (EMSAs)

DNA fragments containing the upstream region from *lcp1* and *lcp3* were prepared by PCR with tetR_lcp1_F/R and lcp3_UP_ F/R primer pairs, respectively (Table 1). The 3′ ends of the probe fragments were labeled with DIG-11-ddUTP using the 2nd generation DIG gel shift kit (Roche), according to the manufacturer’s instructions. Binding reaction was performed at 20 °C for 20 min in a 10-μl reaction mixture containing purified his-tagged ACTI_59620 (ACTI_59620-his) or ACTI_69510 (ACTI_69510-his), 10 nM DIG-labeled probe, 0.1 μg of poly-L-lysine, 5 μg of salmon sperm DNA, 20 mM HEPES (pH 7.6), 1 mM EDTA, 10 mM (NH_4_)_2_SO_4_, 1 mM dithiothreitol, 0.2% (*w*/*v*) Tween 20, and 30 mM KCl. The mixtures were then incubated at 20 °C for 20 min, loaded onto 10% polyacrylamide gels in 0.5× Tris-borate-EDTA buffer. Gel electrophoresis was performed as described previously (Kasai et al. 2010). The labeled DNA was detected using a CSPD detection system (Roche) with a C-DiGit image analyzer (LI-Cor Biosciences).

## Results

### Determination of the genome sequence of strain OR16

To identify the rubber-degrading genes in strain OR16, the genome sequence of this strain was determined. The genomic DNA was sequenced using the MiSeq platform, and the draft genome sequences were de novo assembled to produce 64 scaffolds. Then, the gaps among all contigs were closed by whole-genome sequencing using the PacBio RS II system and a combination of PCR plus Sanger sequencing. The final complete sequence of the OR16 genome consisted of a 9,293,892 bp chromosome with an average GC content of 70.3%. The genome contained 8558 protein-coding sequences (CDSs) and 18 and 65 copies of rRNA and tRNA genes, respectively. This genome was deposited in GenBank under accession number AP019371.1. The 16S rRNA gene sequence of strain OR16 has 96.70% to 95.86% identity with those of the type strains, i.e., *A. utahensis* IFO 13244^T^ (AB037012), *A. philippinensis D*SM 43019 ^T^ (X93187), *A. teichomyceticus* DSM 43866^T^ (AJ865472), *A. rectilineatus* IFO 13941^T^ (AB037010), *A. liguriensis* DSM 43865^T^ (AJ865471), *A. cyaneus* DSM 46137^T^ (X93186), *A. italicus* JCM 3165^T^ (AB048217), *A. palleronii* JCM 7626^T^ (AB048216), and *A. regularis* DSM 43151^T^ (X93188). The whole-genome average nucleotide identity (ANI) values between strain OR16 and *A. missouriensis* 431, *Actinoplanes* sp. SE50/110, *Actinoplanes* sp. N902-109, and *A. friuliensis* DSM 7358 were 87.35%, 85.48%, 84.77%, and 84.36%, respectively. Since the values were less than the same-species threshold (95.0%) (Tong et al. 2015), strain OR16 seemed to be a species distinct from the *Actinoplanes* spp.

### Identification of the *lcp* orthologs in strain OR16

**Fig. 2:**
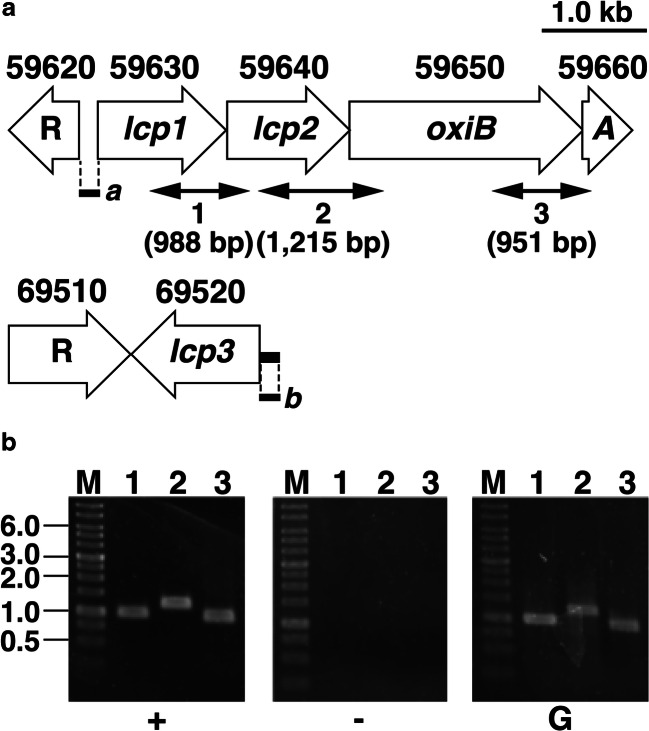
The organization and transcription of the *lcp* gene clusters. **a** Open arrows indicate the genes. Locus tags are indicated above the genes. The putative TATRs-coding genes are indicated by R. Double-headed arrows immediately below the gene cluster diagram indicate the locations of the amplified RT-PCR shown in panel B. Boldface bars *a* and *b* indicate the positions of DNA probes used in EMSA shown in Fig. 5. **b** The results of agarose gel electrophoresis of RT-PCR products obtained with primers targeting 1 (expected size 988 bp), 2 (expected size 1215 bp), and 3 (expected size 951 bp) are shown. The amplified regions and the primer sequences are indicated in panel A and Table 1, respectively. M, molecular size markers; + and -, RT-PCR with and without RT, respectively; G, control PCR with the genomic DNA

A tBLASTn homology search of the genome sequence of OR16 was performed using the amino acid sequence of Lcp (AAR25849) of *Streptomyces* sp. strain K30 as the query, and three homologous genes, ACTI_59630, ACTI_59640, and ACTI_69520, were identified as the most closely related gene sequences; these genes were designated *lcp1*, *lcp2*, and *lcp3*, respectively (Fig. 2a). The deduced amino acid sequences of the *lcp1*, *lcp2*, and *lcp3* gene products shared overall identifies of 56.6%, 53.9%, and 47.6% with that of Lcp from strain K30, respectively. The amino acid sequence identities among biochemically characterized Lcps from actinomycetes are summarized in Table 2. The deduced amino acid sequences encoded by ACTI_59650 and ACTI_59660 exhibited 71.6% and 78.0% identity with those of *oxiB* and *oxiA*, respectively; *oxiB* and *oxiA* are involved in degradation of low-molecular-weight rubber-degrading compounds in *Streptomyces* sp. strain K30 (Rose et al. 2005). In contrast, no other known genes, which may be involved in rubber degradation, were detected in the vicinity of the *lcp3* gene. ACTI_59620 and ACTI_69510, encoding putative TetR/AcrR-type transcriptional regulators (TATRs), were located adjacent to *lcp1* and *lcp3*, respectively (Fig. 2a). Hence, there is a possibility that TATRs seem to be involved in the transcriptional regulation of the rubber-degrading genes.

**Table 2:** Amino acid sequence identity among Lcp proteins in actinomycetes ^*a*^

	Lcp1_OR16_	Lcp2_OR16_	Lcp3_OR16_	Lcp_K30_	Lcp1_VH2_	Lcp2_VH2_	Lcp_SH22a_	Lcp_RPK1_
Lcp1_OR16_	100%	52.1% (213/409)	49.4% (205/415)	56.6% (231/408)	64.4% (266/413)	57.0% (235/412)	59.5% (244/410)	72.4% (296/409)
Lcp2_OR16_		100%	45.7% (189/414)	53.9% (221/410)	49.9% (206/413)	45.3% (186/411)	48.3% (199/412)	52.1% (213/409)
Lcp3_OR16_			100%	47.6% (196/412)	43.0% (177/412)	38.3% (158/413)	42.1% (175/416)	44.6% (185/415)
Lcp_K30_				100%	51.6% (212/411)	46.0% (190/413)	51.8% (213/411)	56.8% (233/410)
Lcp1_VH2_					100%	60.7% (249/410)	62.6% (258/412)	68.2% (279/409)
Lcp2_VH2_						100%	67.3% (276/410)	57.5% (238/414)
Lcp_SH22a_							100%	62.4% (257/412)
Lcp_RPK1_								100%

^a^Lcp_K30_, Lcp from *Streptomyces* sp. K30 (AAR25849); Lcp1_VH2_ and Lcp1_VH2_, Lcps from *G. polyisoprenivorans* VH2 (ABV68923 and AFA76036); Lcp_SH22a_, Lcp from *Nocardia nova* SH22a (WP025350295); Lcp_RPK1_, Lcp from *R. rhodochrous* RPK1 (AMY60409)

To define the operon structure of the *lcp* genes, RT-PCR analysis was performed with total RNA extracted from strain OR16 grown with NR latex. RT-PCR amplification products of the expected size were detected in the intergenic regions of *lcp1*-*lcp2*, *lcp2*-*oxiB*, and *oxiB*-*oxiA* (Fig. 2b). These results suggest that the *lcp1*, *lcp2*, *oxiB*, and *oxiA* genes are organized in the same transcriptional unit.

### Heterologous expression of the *lcp* genes in *E. coli*

**Fig. 3:**
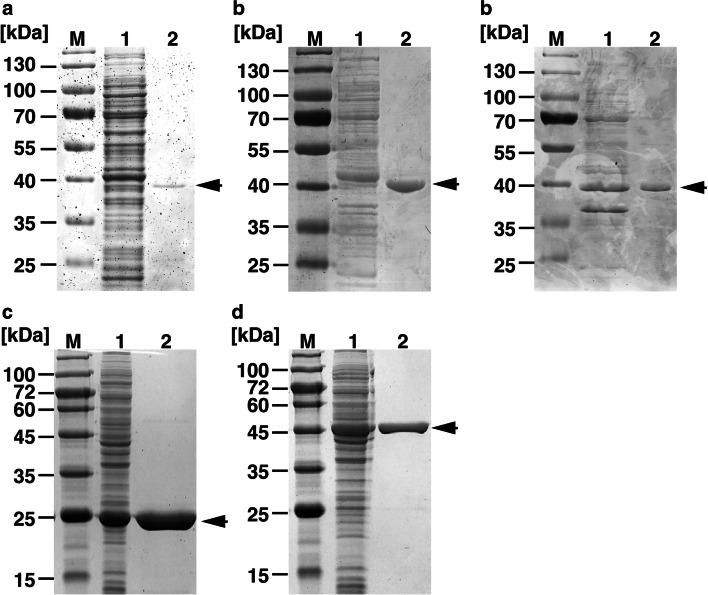
SDS-PAGE analysis of protein fractions. Proteins were separated on a SDS 12% polyacrylamide gel and stained with Coomassie brilliant blue. Lcp1-his, Lcp2-his, and Lcp3-his, ACTI_59620-his, and ACTI_69510-his are shown in panels (**a**), (**b**), (**c**), (**d**), and (**e**), respectively. M, molecular weight markers; 1, crude extract of the *E. coli* cells containing *lcp1*, *lcp2*, *lcp3*, ACTI_59620, or ACTI_69510; 2, purified proteins. Molecular masses are given on the left

To examine the roles of Lcp1, Lcp2, and Lcp3, each gene was fused with a 10× histidine-tag and introduced into *E. coli* Rosetta-gami B(DE3)pLysS cells. When the cell extracts from *E. coli* expressing each gene were prepared and analyzed by SDS-polyacrylamide gel electrophoresis (SDS-PAGE), the presence of proteins of corresponding sizes was observed (Fig. 3a–c). To characterize the activity toward NR latex, each of these his-tagged proteins (Lcp1-his, Lcp2-his, and Lcp3-his) was purified by Ni-affinity column chromatography. When the purified proteins were incubated with NR latex, oxygen consumption activity was observed. No consumption of oxygen was observed without protein or NR latex, strongly suggesting that these enzymes are involved in rubber degradation. The optimal temperature and optimal pH for the oxygen consumption activity of Lcp1-his, Lcp2-his, and Lcp3-his with NR latex were determined to be 30 °C and 7.0, 35 °C and 7.5, and 30 °C and 6.5, respectively. The specific activities of Lcp1-his, Lcp2-his, and Lcp3-his were 4.02 ± 0.65 (30 °C, pH 7.0), 1.17 ± 0.07 (35 °C, pH 7.5), and 0.22 ± 0.01 (30 °C, pH 6.5) U/mg protein, respectively (see Fig. S1 in the supplemental material).

### Transcriptional induction of the *lcp* genes

**Fig. 4:**
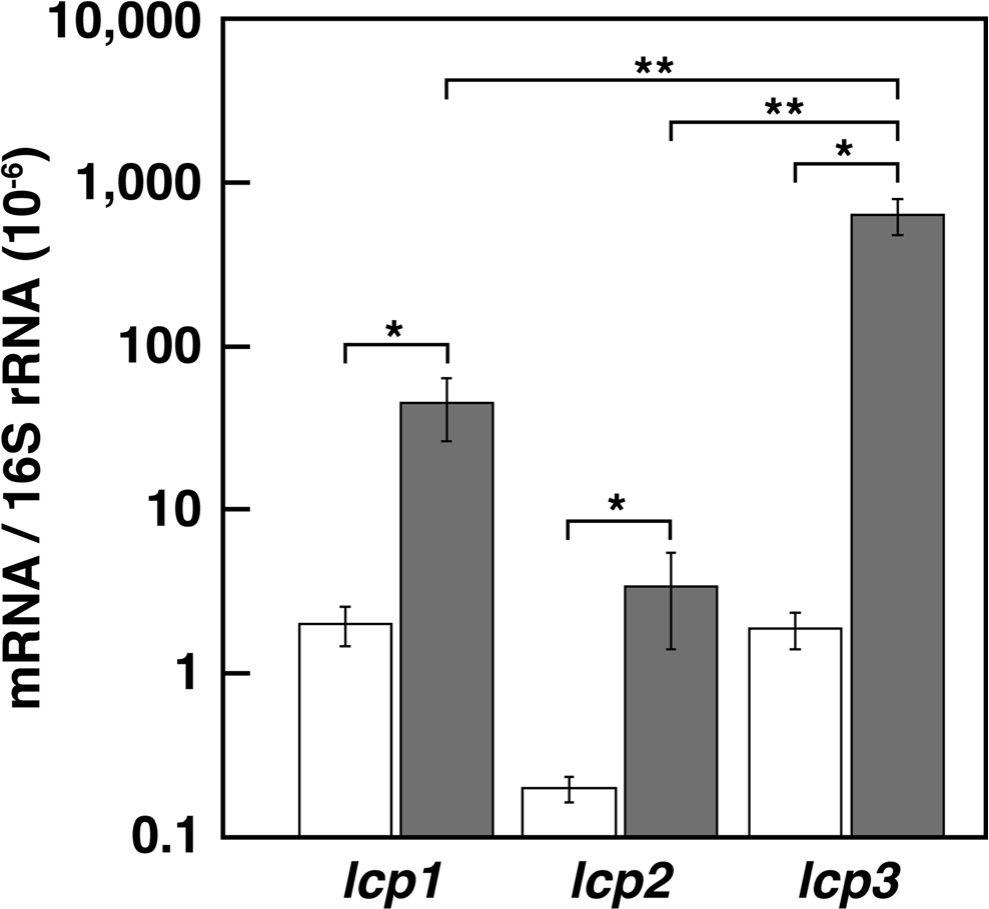
Quantification of the expression levels of the *lcp* genes. Total RNA was isolated from the OR16 cells grown in W medium containing 10 mM succinate without NR (open bars) or with NR (gray bars). The mRNA expression levels were calculated as a ratio of 16S rRNA gene expression. The data are mean values ± standard deviations for four independent experiments. Statistical analysis was performed using Student’s *t* test. *, *P* < 0.05; **, *P* < 0.005

To determine whether transcription of the *lcp* genes is induced in response to NR, the mRNA levels of *lcp1*, *lcp2*, and *lcp3* were measured by quantitative RT-PCR (qRT-PCR) analysis using the total RNA harvested from cells grown on succinate with or without NR latex. Cells grown on succinate exhibited extremely low transcription levels of these three genes. In contrast, the transcription levels of *lcp1*, *lcp2*, and *lcp3* in cells grown with NR latex were 22.2-fold, 17.1-fold, and 335-fold higher, respectively, than those in cells grown without NR latex (*P* < 0.05 by Student’s *t* test) (Fig. 4). These results strongly suggested that the transcription of these *lcp* genes is induced during NR utilization in OR16.

### Binding ability of the regulators of the *lcp* genes

**Fig. 5:**
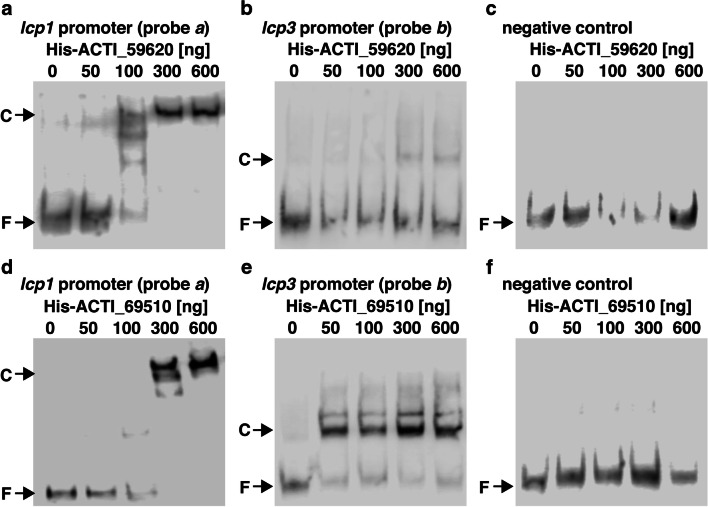
Binding of ACTI_59620-his and ACTI_69510-his to the upstream regions from *lcp1* and *lcp3*. DIG labeled DNA probes (10 nM) were incubated in the presence of purified ACTI_59620-his (**a**–**c**) and ACTI_69510-his (**d**–**f**). DNA fragments including the upstream regions from *lcp1* (probe *a*; A and D) and *lcp3* (probe *b*; B and E) and internal region of 16S rRNA gene of OR16 (negative control; C and F) were used as a probe for EMSA. The size and position of DNA probes *a* and *b* are shown in Fig. 2. Arrows with F and C indicate the positions of the unbound probe (free DNA) and protein-DNA complex, respectively

To confirm the role of the ACTI_59620 and ACTI_69510 gene products in regulating the transcription of the *lcp* genes, these genes were expressed in *E. coli* BL21(DE3) cells as histidine-tagged proteins (ACTI_59620-his and ACTI_69510-his). To characterize the DNA binding ability of ACTI_59620-his and ACTI_69510-his, these proteins were purified to near homogeneity (Fig. 3d and e). Purified ACTI_59620-his and ACTI_69510-his were used in electrophoretic mobility shift assays (EMSAs) with 199-bp and 203-bp DNA probes containing the upstream regions from *lcp1* or *lcp3*, respectively (Fig. 5). As shown in Fig. 5a, ACTI_59620-his was able to bind to the upstream region from *lcp1*. Furthermore, a weakly shifted band was observed when the upstream region from *lcp3* was used as probe (Fig. 5b). The results suggested that ACTI_59620 mainly bound to the promoter of *lcp1* to regulate transcription. In contrast, ACTI_69510-his and DNA complexes were formed when the regions upstream from *lcp1* and *lcp3* were used as probes (Fig. 5d and e). This indicated that ACTI_69510 is able to interact with both promoters of *lcp1* and *lcp3*. Based on these results, the transcriptional induction of the *lcp1* operon seemed to be regulated by ACTI_59620 and ACTI_69510. The regulation of *lcp3* transcription mainly requires the binding of ACTI_69510 to the promoter.

## Discussion

Multiple *lcp* homologs have rarely been identified in rubber degraders such as *G. polyisoprenivorans* VH2. The *lcp1* and *lcp2* genes are located on the chromosome and on the plasmid of VH2, respectively (Hiessl et al. 2012). On the other hand, *lcp1*, *lcp2*, and *lcp3* are all located on the chromosome in OR16. In particular, the *lcp1* and *lcp2* genes are tandemly located in the same orientation (Fig. 2a). The organization of the *lcp* genes is similar to that of the *lcp* genes in *Streptomyces* sp. strain CFMR7 (Nanthini et al. 2017). The deduced amino acid sequences of the *lcp1*, *lcp2*, and *lcp3* gene products of strain OR16 have 63.3%, 60.0%, and 59.2% identity, respectively, with those of the corresponding *lcp* genes from strain CFMR7. Hence, the *lcp* genes of strain CFMR7 might be involved in the rubber utilization as well as those of strain OR16. Because Lcp was predicted to be secreted via the twin arginine translocation (Tat) pathway (Hiessl et al. 2012; Yikmis et al. 2008), it is critical to identify the Tat signal sequences in Lcp1, Lcp2, and Lcp3. In OR16, the RRxxLx motifs of Lcp1, Lcp2, and Lcp3 were identified between the 6th and 10th, 3rd and 7th, and 6th and 10th residues, respectively. The putative cleavage sites of the Tat signal sequences, which contain the AxA motifs of Lcp1, Lcp2, and Lcp3, were located between 30th and 31st, 26th and 27th, and 33rd to 34th residues, respectively. Therefore, the *lcp* gene products might be secreted via the Tat pathway in strain OR16.

When NR was incubated with purified Lcps, the oxygen consumption activity was observed. The enzymatic activities are comparable with those of other reported Lcps in strain K30 (4.6 U/mg), strain VH2 (1.3 U/mg), and *R. rhodochrous* RPK1 (3.1 U/mg) (Birke et al. 2015; Hiessl et al. 2014; Watcharakul et al. 2016). Therefore, these Lcps might be involved in the rubber degradation by OR16 (Fig. 1). The activity of Lcp1 was approximately 7 times and 18 times higher than those of Lcp2 and Lcp3, respectively, suggesting that Lcp1 is mainly involved in rubber degradation in strain OR16. Unlike the enzymatic activities of the Lcp enzymes, the transcription level of *lcp3* is significantly higher than those of *lcp1* and *lcp2* (*P* < 0.005 by Student’s *t* test). According to their transcription levels, the role of *lcp3* is thought to also be important for the rubber degradation.

RT-PCR analysis revealed that *oxiB* and *oxiA* homologous genes (ACTI_59650, ACTI_59660) tandemly located in the downstream region from *lcp2* were cotranscribed with the *lcp1* and *lcp2* genes during rubber utilization. It has been reported that rubber degradation intermediate, low-molecular-weight aldehyde compounds are further metabolized by *oxiAB*, encoding oxidoreductase, in *Streptomyces* sp. strain K30 (Rose et al. 2005). It was suggested that ACTI_59650 and ACTI_59660 are involved in the catabolism of aldehyde intermediates for the utilization of rubber by strain OR16.

The ACTI_59620 gene encodes a putative TATR and is located upstream from *lcp1* in the opposite direction (Fig. 2a). The deduced amino acid sequence of this gene product is similar to those of TATRs from *Rhodococcus* sp. Q15 (AAK97457) and *R. opacus* B-4 (BAH50508). It has been reported that TATRs regulate the transcription of genes involved in antibiotic resistance, antibiotic biosynthesis, catabolic pathways, and biofilm formation in Gram-negative and Gram-positive bacteria as a repressor or an activator (Pompeani et al. 2008; Ramos et al. 2005). Based on these facts, ACTI_59620 seemed to be involved in the regulation of *lcp* transcription. Furthermore, ACTI_69510, located adjacent to *lcp3* and encoding a putative TATR, was found (Fig. 2a). ACTI_69510 seemed to be involved in *lcp3* transcription. Interestingly, the amino acid sequences of the N-terminal (residues 22 to 196) and C-terminal (residues 201 to 401) regions of ACTI_69510 exhibited 25.0% identity with each other. In addition, a TATR superfamily-specific domain was found in each region, suggesting that ACTI_69510 was formed by duplication of the regions.

LcpRB_A3(2)_ and LcpR_VH2_ were identified as transcriptional regulators for putative *lcp* gene expression in *S. coelicolor* A3(2) and *G. polyisoprenivorans* VH2, respectively (Coenen et al. 2019; Oetermann et al. 2019). These promoter regions of *lcp1*_VH2_ and *lcp2*_VH2_, which interacted with LcpR_VH2_, contained palindromic sequences, 5′-GATGTTACAACGTTACTCGCGTTGTTACATC-3′ and 5′-GATACAGAGAAGCATAGACTGTAACTCGGTTTC-3′, respectively. In the binding region of ACTI_59620, a palindromic sequence (5′-GATGCGAATTTGTAACAGCGTATCAGCAATC-3′), which is similar to the sequence found in the *lcp1*_VH2_ promoter region, was identified. Due to the amino acid sequence similarity (48.7% identity) between LcpR_VH2_ and ACTI_59620, the formation of a protein-DNA complex by ACTI_59620 requires the palindromic sequence, similar to LcpR_VH2_.

The amino acid sequence of ACTI_69510 shared 72.3% identity with that of LcpRB_A3(2)_, which binds to the promoter region of *lcp*_A3(2)_ in strain A3(2) (Coenen et al. 2019). It has been reported that the LcpRB_A3(2)_-binding sequence contains a palindromic sequence (5′-TATGTTAATGAAAAATCACA-3′). In the upstream region from *lcp3*, a similar sequence (5′-TATGTTAATGGAAAATCACA-3′) was found. Therefore, the palindromic sequence seemed to be involved in the binding of ACTI_69510. Surprisingly, ACTI_69510 bound not only to the *lcp3* promoter but also to the *lcp1* promoter. However, a palindromic sequence similar to the palindromic sequence in the *lcp3* promoter was not detected in the upstream region from *lcp1*. Because several palindromic sequences were identified in the *lcp1* promoter region, the other sequence seemed to be involved in ACTI_69510 binding. Because ACTI_69510 has two helix-turn-helix DNA-binding motifs that are similar to each other (31.9% identity), ACTI_69510 might be able to recognize several palindromic sequences, including the *lcp1* and *lcp3* promoters, for its binding. The broad binding specificity of ACTI_69510 is thought to be effective in inducing the transcription of the *lcp* genes and reasonable for the efficient utilization of rubber in strain OR16.

In this study, we identified the rubber-degrading genes of strain OR16. This strain possesses three *lcp* genes which might be required for the utilization of natural rubber. Furthermore, two TATRs that have the ability to bind to the promoter regions of the *lcp* genes were identified. Further analysis including deletion of the TATR-coding genes and the *lcp* promoter assay will enable us to clarify the actual function and contribution of these TATRs to the transcriptional regulation of rubber-degrading genes.

## Electronic supplementary material

ESM 1 (PDF 503 kb)
