# Supplementary material for: Characterization of the genes responsible for rubber degradation in Actinoplanes sp. strain OR16
Source: Appl Microbiol Biotechnol. 2020 Jul 18;104(17):7367–76. doi: 10.1007/s00253-020-10700-1 (PMC7413915; doi:10.1007/s00253-020-10700-1)
Supplement: Supplementary file 1 — (PDF 503 kb) [file 253_2020_10700_MOESM1_ESM.pdf]

Applied Microbiology and Biotechnology

Characterization of the Genes Responsible for Rubber Degradation in *Actinoplanes* sp. Strain OR16.

Namiko Gibu [1], Tomoka Arata [1], Saya Kuboki [1], Dao Viet Linh [1#], Masao Fukuda [1$], Alexander Steinbüchel [2,3], Daisuke Kasai [1*]

[1]*Department of Bioengineering, Nagaoka University of Technology, Nagaoka, Japan,* [2]*Institut für Molekulare Mikrobiologie und Biotechnologie, Westfälische Wilhelms-Universität Münster, Münster, Germany.,* [3]*Environmental Science Department, King Abdulaziz University, Jeddah, Saudi Arabia.*

[*]Corresponding author: Daisuke Kasai
Department of Bioengineering
Nagaoka University of Technology
Nagaoka, Niigata 940-2188, Japan
E-mail: dkasai1@vos.nagaokaut.ac.jp

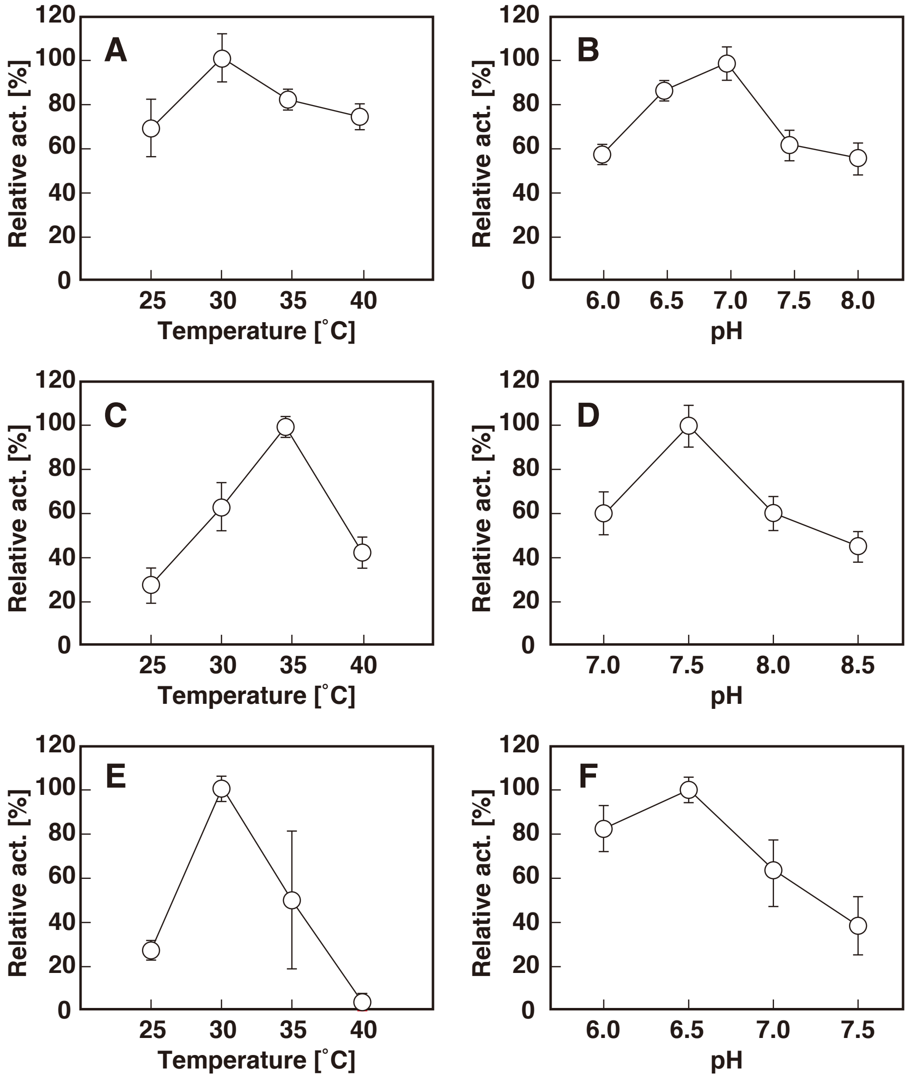

Fig. S1. Effect of temperature (A, C, and E) and pH (B, D, and F) on Lcp activity. Relative oxygen consumption activities of Lcp1-his (A and B), Lcp2-his (C and D), and Lcp3-his (E and F) were indicated. Each value is the average ± standard deviation of three independent experiments performed in parallel.
